# Multi-replicas integrity checking scheme with supporting probability audit for cloud-based IoT

**DOI:** 10.7717/peerj-cs.1790

**Published:** 2024-01-16

**Authors:** Yilin Yuan, Fan Yang, Xiao Wang, Yimin Tian, Zichen Li

**Affiliations:** 1 College of Information Engineering, Beijing Institute of Graphic Communication, Beijing, China; 2 Guangdong Provincial Key Laboratory of Novel Security Intelligence Technologies, Guangzhou, Guangdong, China; 3 Department of Information Science and Technology, Tianjin University of Finance and Economics, Tianjin, China; 4 College of Intelligence and Computing, Tianjin University, Tianjin, China; 5 Science Education Department, Beijing Institute of Graphic Communication, Beijing, China

**Keywords:** Multi-replicas integrity verification, Public auditing, EHR data, Identity-based encryption, Probability audit

## Abstract

Nowadays, more people are choosing to use cloud storage services to save space and reduce costs. To enhance the durability and persistence, users opt to store important data in the form of multiple copies on cloud servers. However, outsourcing data in the cloud means that it is not directly under the control of users, raising concerns about security and integrity. Recent research has found that most existing multicopy integrity verification schemes can correctly perform integrity verification even when multiple copies are stored on the same Cloud Service Provider (CSP), which clearly deviates from the initial intention of users wanting to store files on multiple CSPs. With these considerations in mind, this paper proposes a scheme for synchronizing the integrity verification of copies, specifically focusing on strongly privacy Internet of Things (IoT) electronic health record (EHR) data. First, the paper addresses the issues present in existing multicopy integrity verification schemes. The scheme incorporates the entity Cloud Service Manager (CSM) to assist in the model construction, and each replica file is accompanied with its corresponding homomorphic verification tag. To handle scenarios where replica files stored on multiple CSPs cannot provide audit proof on time due to objective reasons, the paper introduces a novel approach called probability audit. By incorporating a probability audit, the scheme ensures that replica files are indeed stored on different CSPs and guarantees the normal execution of the public auditing phase. The scheme utilizes identity-based encryption (IBE) for the detailed design, avoiding the additional overhead caused by dealing with complex certificate issues. The proposed scheme can withstand forgery attack, replace attack, and replay attack, demonstrating strong security. The performance analysis demonstrates the feasibility and effectiveness of the scheme.

## Introduction

With the advent of the era of big data, the types and quantities of data have shown explosive growth. At the same time, the methods and devices of data storage have also received more attention. For example, from large-capacity nonportable solid-state storage devices to small-capacity portable USB flash drives, to large-capacity portable mobile hard drives, people are always willing to store data on devices with high flexibility and capacity. Fortunately, cloud storage services can better meet the needs of users. Users who choose to use cloud storage services do not need to deploy any physical devices locally, nor do they need to be involved in the daily maintenance of outsourcing data, they can simply focus on enjoying the service. Therefore, cloud storage services have been chosen by more and more users in recent years. However, users who choose cloud storage services will, by default, transfer the control of the data to the Cloud Storage Provider (CSP) after uploading the outsourced file. Despite the popularity of cloud storage services, their security and reliability remain subject to skepticism. Therefore, ensuring the security and integrity of outsourced data when using cloud storage services is a research hotspot for scholars. Currently, more valuable schemes have been proposed that can effectively verify the integrity of remote data.

To enhance the availability and durability of the outsourced data, users choose to store important data on multiple CSPs with different geographic locations or different types. Therefore, after completing the data upload, verifying the integrity of duplicate files is an issue worth considering. On the one hand, due to the increased complexity of verifying multicopy files compared to a single file, the following issues need to be considered: (1) How should the duplicate files be generated to guarantee storage security? (2) How to design the homomorphic verification tag (HVT) to realize synchronous verification of duplicate file integrity? (3) How can we improve verification efficiency? (4) How do we implement recovery for damaged replicas? These are the primary issues that need to be addressed when designing a multicopy data integrity verification scheme. On the other hand, most existing multicopy file integrity verification schemes have almost not taken into account the distribution of replica storage locations. Specifically, while most schemes claim to simultaneously check replicas stored in different geographical locations, this is not actually the case, as the duplicate files in their schemes are actually stored on the same CSP (which will be detailed in the ‘Related Work’ section). Clearly, if the storage server fails, all duplicate files of the user will be damaged. Even if the cloud service provider offers compensation, the user’s important data has already been compromised, which is bound to erode the user’s confidence in them. Therefore, the user’s duplicate files should be stored on multiple CSPs located in different geographical locations to minimize the risk of data loss. Similarly, how to conduct synchronous checks on these duplicate files is also a crucial issue.

As mentioned earlier, the era of big data has arrived and both storage and privacy security should be guaranteed for outsourced cloud data. The Internet of Things (IoT), as a rapidly evolving technology in recent years, connects devices and sensors through networks, utilizing cloud computing to process and transmit data to achieve interconnectedness of all things. Recently, the proliferation of wearable devices has made the integration of IoT and big data in healthcare even more closely intertwined. For example, most hospitals currently use electronic health records (EHR), which serve as a form of healthcare big data encompassing a patient’s entire life process, including identity information, health status, and medical history, among other details. The EHR data comes from multiple channels, making it comprehensive and detailed. Due to its sheer volume, storing it in the cloud is a viable solution. However, an EHR contains various sensitive information, and outsourcing it directly to the cloud would inevitably lead to privacy breaches. Furthermore, not all EHR data can be shared among hospitals, so creating copies of EHR when patients visit different hospitals would be more convenient. Therefore, as one of the data types in cloud-based IoT, it is essential to safeguard the security and integrity of EHR replica files.

### Related work

To verify remote data integrity in the cloud storage environment, existing schemes can be broadly categorized into two types: data possession verification and data retrieval verification. In 2007,  Ateniese et al. (2007) proposed the Provable Data Possession (PDP) scheme. The PDP scheme employs random sampling and is essentially a probabilistic detection model. Notably, it not only enables blockless verification but also significantly reduces the I/O overhead during the remote checking process. In the same year, Juels & Kaliski (2007) proposed the Proof of Retrievability (PoR) scheme. PoR scheme adds a special data block named “Sentinels” for detection and introduces erasure coding technology, so it can complete remote data integrity checking and data retrieval simultaneously. Building on the PoR scheme,  Shacham & Waters (2008) proposed an enhanced scheme. In Shacham & Waters (2008), two methods for constructing homomorphic verification tag (HVT) are presented: when constructed based on pseudo-random functions (PRF), this scheme supports private verification and is shown to be secure in the standard model; when constructed using BLS signatures, this scheme supports public verification and is proven secure in a random oracle model. Building on these foundational schemes, subsequent research has made significant contributions to the field of data integrity verification (Ateniese et al., 2008; Erway et al., 2009; Wang et al., 2011b; Wang et al., 2011a; Tian et al., 2015; Li, Yan & Zhang, 2020; He et al., 2021; Shu et al., 2021; Wang, Wang & He, 2021; Zhang et al., 2020; Shang et al., 2021).

In the scheme of using public key infrastructure (PKI) to distribute keys, PKI is an indispensable entity. However, the presence of certificates places a substantial burden on verification processes. For example, during data integrity check, the users must verify both the data and the certificate, while the system is tasked with tasks such as certificate generation, forwarding, storage, checking, and updates. In actual use, certificate management will be laborious and inefficient. In 1984, Shamir (1984) proposed an identity-based key system, in which the user’s unique identity, such as e-mail, phone number, *etc*., can serve as a public key and the corresponding private key is generated by the private key generator (PKG). This eliminates the need for PKI, greatly reducing the reliance on certificates in Identity-Based Encryption (IBE). In 2001, Boneh & Franklin (2001) provided the first practical IBE scheme based on the weil pairing. Following this,  Wang et al. (2014) proposed the first public data integrity verification scheme constructed using IBE. Zhang & Dong (2016) proposed a public auditing scheme that combines bilinear mapping and IBE construction, requiring only constant-level computational cost. Tian, Gao & Chen (2019) applied the ideal lattice based on the polynomial structure to key generation and proposed the scheme that can achieve efficient key generation and low-cost storage. Wang, He & Tang (2016) introduce Proxy, a trusted entity, and discuss how to conduct public auditing when users face restrictions on accessing CSPs. Shen et al. (2018) discussed that in the context of a big data environment, by adding a trusted entity Sanitizer, the purpose of hiding sensitive user information is achieved. Li et al. (2017) provide a method to convert the feature vector generated by the biometric information of the users, such as the iris, fingerprint, *etc*., into a usable key and construct a public audit scheme that supports the input of fuzzy identities. Yu et al. (2016) proposed a new method for key construction using RSA.

To address the challenge of public auditing of multiple-replica, Curtmola et al. (2008) introduced the initial multiple replica provable data possession (MR-PDP) scheme. This scheme employs RSA to construct the HVT, demonstrating that the time required to verify multiple copies of files together is significantly less than the time required to verify them individually. However, the calculation and communication cost of this scheme is relatively large. Shamir (1984) designed the HVT by assisting with the vector dot product and proposed a flexible multiple replica provable data possession (FMR-PDP) scheme. Although the FMR-PDP scheme has great advantages in computing and communication overhead, it only considers private verification, which limits its practicality. Barsoum & Hasan (2014) proposed a Provable Multicopy Dynamic Data Possession (PMDDP) scheme to realize replica dynamics by mapping version tables. The PMDDP scheme nests the number of copies into the HVT constructed by RSA. Although the modification, insertion, and deletion of the specified data block in the copy file can be completed, if the verification fails, the current integrity verification will inevitably fail and one cannot locate the corrupted copy. Furthermore, Hou, Yu & Hao (2018) devised a scheme that utilizes algebraic signatures to construct HVT and facilitate replica dynamics. Long, Li & Peng (2019) proposed applying chaotic mapping to the construction of full-node AVL trees to achieve replica dynamics. Wei et al. (2016) proposed to use fully homomorphic encryption (FHE) to generate multicopy files. Furthermore, Zhang et al. (2016) and Guo et al. (2020) independently proposed a public auditing scheme using the Merkle tree to achieve replica dynamics. Zhou et al. (2020) formalized a dynamic multicopy authentication scheme constructed using certificateless cryptography. To complete the unified management of multiple CSPs,  Wang (2014) introduced an entity Combiner, which can transfer information between multiple CSPs and TPAs during the audit process. Likewise, Li, Yan & Zhang (2021) introduced the Cloud Organizer entity to achieve similar functions. Additionally, to facilitate dynamic operations on replicas, Zhang et al. (2021) combined a Merkle tree with a B+ tree to construct an IBM tree. Zhou et al. (2020) achieved dynamic data manipulation using certificateless signatures coupled with table structures and Merkle hash trees. Yi, Wei & Song (2017) focused on the generation of replica files using fully homomorphic encryption, while Peng et al. (2019) contemplated the construction of compressed identity arrays as a homomorphic verification substitute for replicas.

### Motivation and contribution

**Figure 1:**
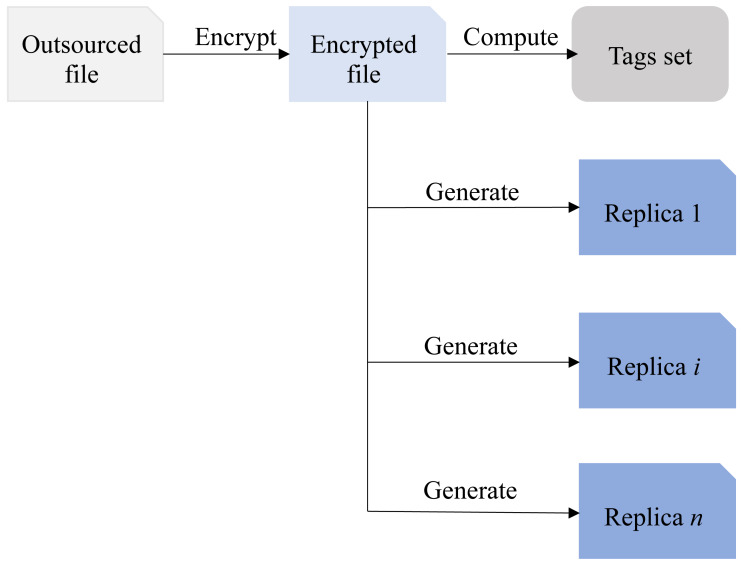
The relationship between encrypted file, duplicate files and tags set.

In the MR-PDP scheme, the user first encrypts the outsourced file, and then utilizes the encrypted file to generate multiple replicas and tags set, respectively. These duplicate files along with their respective tag sets are subsequently uploaded to the CSP by the user. This approach has been adopted by the references Curtmola et al. (2008), Li, Yang & Wu (2017), Barsoum & Hasan (2014), Hou, Yu & Hao (2018), Long, Li & Peng (2019) and Wei et al. (2016). As shown in Fig. 1, the relationship between encrypted file, duplicate files, and tags set is illustrated.

As Fig. 1 demonstrated, the tags set is derived from the encrypted file and remains independent of the content and quantity of the replica files. Clearly, using this method can greatly reduce computational overhead, especially when dealing with a large number of replicas. Although cloud service providers claim to send replica files and the tag set <*T*, *F*_*i*_ > to multiple CSPs, even if all the content is sent to the same CSP, it will not affect the normal execution of subsequent data integrity verification. However, if the CSP storing all replica files experiences an outage, the user’s cloud replicas will be lost completely, and it will not even be possible to recover the damaged replicas with the help of other replica files, the consequences would be disastrous. Hence, when designing an integrity verification scheme involving multiple replicas, precautions must be taken to prevent the cloud service operator from storing all replicas on the same CSP to avoid irreparable losses to the users.

Based on the above considerations, in this article, we focus on the EHR data and aim to solve the multi-replica synchronization integrity verification problem. The contributions are summarized as follows: (1) We employ identity-based encryption (IBE) to generate the private key and then construct HVT, effectively bypassing the overhead of public key certificates. We combine symmetric encryption and masking technology to generate duplicate EHR files. This method can keep storage safe and enable bad block recovery in the event of replica corruption. (2) Considering that duplicate EHR files are stored in multiple CSPs with diverse geographical locations, therefore, in our proposal, we introduce a crucial entity known as the Cloud Server Manager (CSM) that can act as a ‘bridge’ between the Patient and various CSPs. The CSM allocates storage servers for multiple copies of the Patient EHR and records the allocation results in the storage distribution table (SDT). In the public auditing phase, the CSM transmits the integrity challenge initiated by the TPA to multiple CSPs and then aggregates the audit proofs returned by the multiple CSPs. However, due to irresistible factors such as channel delay, CSPs in different geographical locations may experience delays in returning audit proof in time. Thus, to ensure the practical implementation, our proposal supports probability audit and provides a specific description. (3) Since the CSPs are untrusted, under the given security model, our proposal can effectively resist forgery attack, replace attack, replay attack, and collusion attack. Lastly, the performance evaluation section validates the feasibility and effectiveness of our scheme.

The remaining sections of this paper are arranged as follows. Preliminaries introduce the system model, design model, notations, and cryptographic knowledges. The next section presents the system components and the security model. The probability audit section describes the auxiliary data structures including the storage distribution table, fault tolerance value, and result record table. Following that, we provide a detailed description of the proposal. Subsequently, the paper presents the security analysis and performance evaluation sections. The final section concludes this paper.

## Preliminaries

### System model

**Figure 2:**
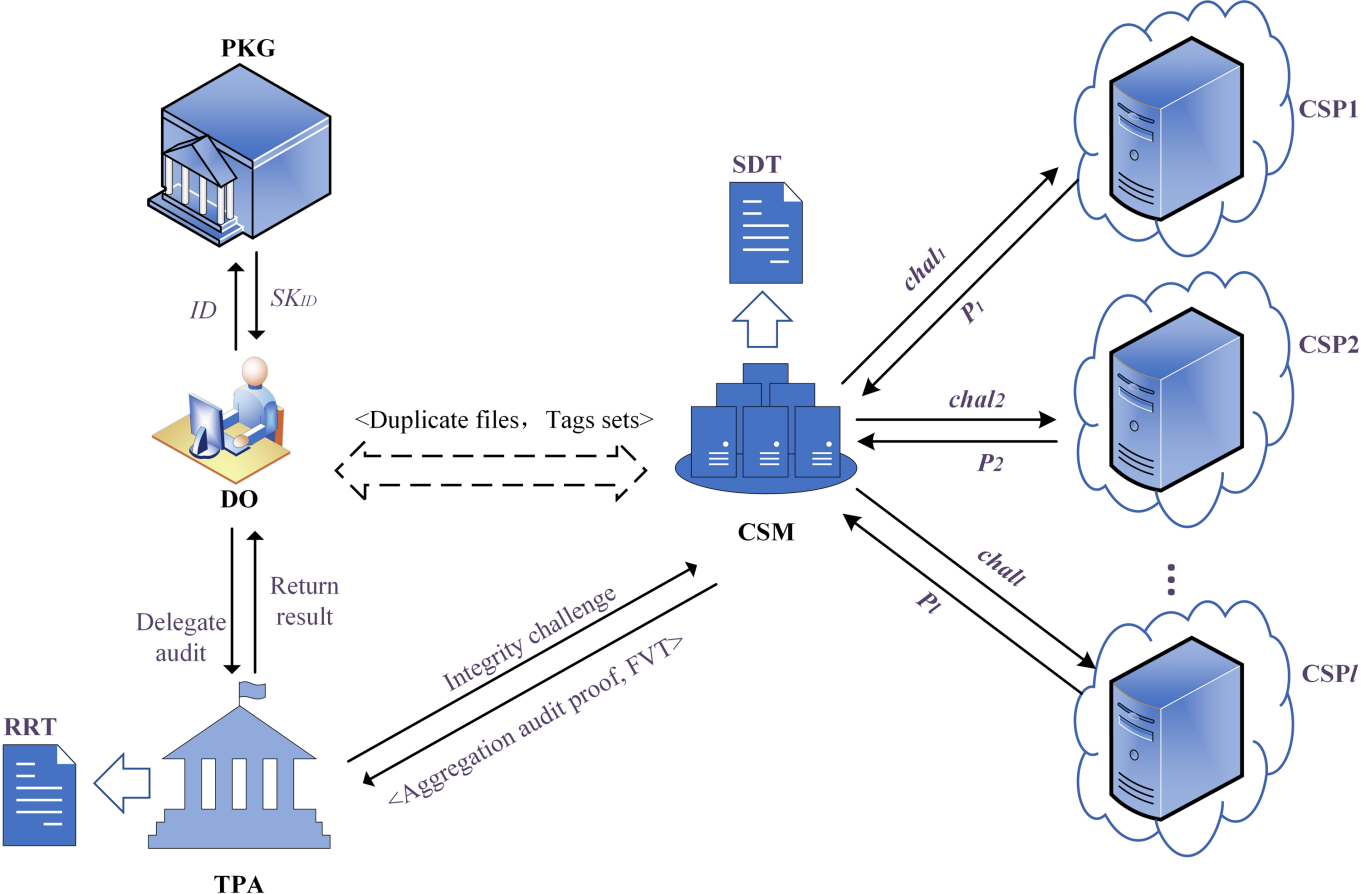
The system model diagram.

Our proposal consists of five entities, and the model is shown in Fig. 2. (1) Patient: considering the sensitivity and importance of EHRs data, the Patient produces multiple replicas and uploads them to multiple CSPs in diverse geographical locations and types. The Patient expects that the security and integrity of the replicas can be guaranteed. (2) Cloud Service Manager (CSM): our proposed scheme introduces an important and indispensable entity named CSM, which is equivalent to the ‘intermediary’ between TPA and multiple CSPs. It allocates storage servers for replica files of the Patient, transmits the integrity challenge launched by the TPA to the multiple CSPs, and aggregates the audit proofs returned by the CSPs. (3) Cloud Service Provider (CSP): the untrusted entity that provides the Patient with data storage services. In the public auditing phase, the CSPs will respond to the integrity challenge initiated by the TPA, calculate, and return the audit proof to the CSM. (4) Private Key Generator (PKG): the trusted entity that generates a reliable private key for the Patient according to its unique identifier. (5) Third-Party Auditor (TPA): the trusted entity performing remote data integrity checks for duplicate files of the Patient after obtaining the authorization.

### Design model

Our proposal should achieve the following goals:

(1) **Correctness**: the correctness should include private key correctness and audit correctness.

(a) **Private key correctness**: the private key generated by the PKG will only be accepted after successfully passing the Patient’s correctness verification.

(b) **Audit correctness**: the correctness of the aggregation audit proof returned by the CSM can be verified by the TPA. Note that if the FVT returned by the CSM is invalid, the TPA will abort the integrity checking and notify the Patient immediately.

(2) **Resist forgery/replace/replay attack**: our proposal can effectively resist forgery/replace/repay attack.

(3) **Support probability audit**: our proposal supports probabilistic detection based on guaranteeing the storage security of duplicate files.

### Notations

We give the notations used in the description of our scheme in Table 1.

**Table 1:** Notations and descriptions. Symbols used in full text and their descriptions.

Notation	Meaning
*p*	One large prime
*G*_1_, G_2_	Multiplicative cyclic groups
*g*	A generator of group *G*_1_
*e*	A bilinear map: *e*:*G*_1_ × *G*_1_ → *G*_2_
${Z}_{P}^{\ast }$	A prime field with nonzero elements
*H*, *H*_1_, *H*_2_	Cryptographic hash function
*pp*	The system public parameter
*ID*	The group user’s identity
*SK*_*ID*_	The group user’s private key
*msk*_*ID*_, *mpk*_*ID*_	The master secret key and master public key
*F*	The Patient’s encrypted outsourced file
*n*	The number of replicas
*s*	The number of the CSP
*σ*_*i*_	The *i*-th block’s tag, 1 ≤ *i* ≤ *n*

### Cryptographic knowledge

**(1) Bilinear maps**

Let *G*_1_, *G*_2_ be multiplicative cyclic groups with the order *p*, *g* is a generator of *G*_1_. A bilinear map *e*:*G*_1_ × *G*_1_ → *G*_2_ satisfies the following properties:

(a) Bilinearity: ∀*u*, *v* ∈ *G*_1_ and $\forall a,b\in {Z}_{P}^{\ast }$, *e*(*u*^*a*^, *v*^*b*^) = *e*(*u*, *v*)^*ab*^;

(b) Non-degeneracy: *e*(*g*_1_, *g*_2_) ≠ 1;

(c) Computable: there is an efficient algorithm to calculate *e*.

**(2) Security assumptions**

**Computational Diffie-Hellman assumption**. For unknown $\forall a,b\in {Z}_{P}^{\ast }$, given *g*, *g*^*a*^ and *g*^*b*^ as input, output *g*^*ab*^ ∈ *G*_1_.

**Definition 1** **(CDH assumption)**. The advantage of a *PPT* (probabilistic polynomial time) algorithm $\mathcal{A}$ in solving the CDH problem in *G*_1_ defined below is negligible:

\begin{eqnarray*}AdvCD{H}_{\mathcal{A}}=\Pr\nolimits [\mathcal{A}(g,{g}^{a},{g}^{b})={g}^{ab}:a,b\leftarrow ^{R}{Z}_{P}^{\ast }]. \end{eqnarray*}

## System Components and Security Model

### System components

Our proposed scheme consists of nine algorithms: Setup, KeyGen, ReplicaGen, TagGen, Challenge, ProofGen, ProofAgg, ProofVerify, Compensation. Each algorithm is described as follows.

***Setup*** (1^*k*^) → (*pp*, *mpk*, *msk*) is the “System Initialization” algorithm run by the PKG. It takes the security parameter *k* as input and outputs the system public parameter *pp*, the master public key *mpk* and the master secret key *msk*.

**KeyGen** (*pp*, *mpk*, *msk*, *ID*) → *SK*_*ID*_ is the “Private Key Generation” algorithm run by the PKG. It takes the system public parameter *pp*, the master public key *mpk*, the master secret key *msk* and Patient’s identifier *ID* as input, and outputs the private Patient key *SK*_*ID*_.

***ReplicaGen*** *F*′ → 𝔽 is the “Replica Files Generation” algorithm run by the Patient. It takes the outsourced EHR file as input and outputs the cloud duplicates.

***TagGen*** (𝔽, *pp*, *mpk*, *SK*_*ID*_) → 𝕋 is the “Tags Set Generation” algorithm run by the Patient. It takes the duplicate files 𝔽, the system public parameter *pp*, the master public key *mpk* and the private Patient key *SK*_*ID*_ as input, and outputs the tags set for each replica. Then, the Patient sends duplicates 𝔽 and all tags sets 𝕋 to the CSM and delete the local storage. Following this, the CSM verifies the accuracy of all tag sets. Upon successful verification, it proceeds to allocate them and upload them to the storage server. Subsequently, the CSM records the allocation results in the Storage Distribution Table (SDT).

***Challenge*** is the “Launch Integrity Challenge” algorithm run by the TPA. The TPA periodically generates the integrity challenge *chal* for multiple copies and sends them to the CSM. Upon received, the CSM searches the SDT and transmits integrity challenge set to the corresponding CSPs.

***ProofGe*n** is the “Audit Proof Generation” algorithm run by the CSPs. The CSPs receive the challenge message, compute, and respond to the audit proofs to the CSM.

***ProofAgg*** is the “Audit Proof aggregation” algorithm run by the CSM. After receiving the responses, the CSM counts the number of audit proofs, sets the fault tolerance value (FTV) *ξ*, calculates the aggregation audit proof *P*_*agg*_, and then sends *P*_*agg*_, *ξ* to the TPA.

***ProofVerify*** is the “Audit Proof Verification” algorithm run by the TPA. After receiving the response from the CSM, the TPA searches the Result Record Table (RRT) to judge the validity of the FVT. Once the FVT is illegal, or the check fails, the TPA aborts and notifies the Patient. Otherwise, the TPA checks the correctness of the aggregation audit proof *P*_*agg*_.

***Compensatio*n** is the “Claim Compensation” algorithm. The loss or leakage of sensitive information from the EHR cannot be tolerated, and the Patient claims compensation from the cloud service operator after receiving a negative notification.

**Remark 1:** The responsibilities of the CSM are outlined as follows: (a) validate the correctness of the tags set for each replica file. Only after successful verification, the CSM allocates the storage servers for all copy files and documents the outcomes in the SDT. (b) Upon receiving the integrity challenge launched by the TPA, consult the SDT and forward it to the CSPs. (c) Compute the aggregation audit proof according to the audit proofs and the FVT returned by the CSPs and then reply to the TPA.

### Security model

In our proposal, untrustworthy CSPs may launch the following three types of attack models.

(1) **Forgery attack**: During the public auditing phase, if the data block in the replica file stored on the CSP has been damaged due to the CSP’s misbehavior, and this corrupted data block is just challenged, then the CSP has to forge this data block and its corresponding tag to pass the TPA’s integrity verification.

(2) **Replace attack**: During the public auditing phase, if the data block in the replica file stored on the CSP has been damaged due to the CSP’s misbehavior, and this corrupted data block is just a challenge, then the CSP has to replace this data block and its corresponding tag with another intact one to pass the TPA’s integrity verification.

(3) **Replay attack**: During the public auditing phase, if the data block in the replica file stored on the CSP has been damaged due to the CSP’s misbehavior, and this corrupted data block is just a challenge, then the CSP returns the audit proof that has been previously checked to pass the TPA’s integrity verification.

## Probability Audit

To realize probabilistic auditing, the proposed scheme should incorporate some auxiliary data structures, which are described in this section.

### Storage distribution table

Since duplicate EHR files are stored on multiple CSPs, the CSM should maintain a storage distribution table (SDT) locally for easy storage management. One Patient corresponds to one SDT, which is used to record the storage servers of each replica. The SDT consists of three columns and its structure is illustrated in Table 2. Replica number (*RN*) indicates the serial number, where *n*(1 ≤ *i* ≤ *n*) is the number of the copy files. File identifier (F*id*) is the replica identifier. Storage location (*SL*) indexes the storage location, where *l*(1 ≤ *l* ≤ *s*) is the number of storage servers.

**Remark 2:** Take <*RN*_*i*_, *Fid*_*i*_, *SL*_*l*_ > as an example to explain the usage of the SDT. *RN*_*i*_ denotes the *i*th replica, *Fid*_*i*_ is the copy identifier, and *SL*_*l*_ records the storage location. The CSM will assign the CSP to the successfully verified replica and record the result in the SDT.

### Fault tolerance value

During the public auditing phase, the TPA executes the challenge-response protocol and launches replica integrity verification. Upon receiving the message from the TPA, the CSM searches the SDT, dispatches the integrity challenge *chal* to the CSPs, initiates a countdown *cd*, and awaits the return of the audit proofs. However, due to the dispersed geographical locations of the CSPs and variations in channel transmission performance, the response times of different CSPs may differ significantly. Consider the following scenario. The CSM transmits the integrity challenge *chal* to *s* CSPs and starts a countdown *cd*. A geographically distant, yet responsive, CSP promptly computes and returns the audit proof upon receiving the challenge. However, due to factors like channel transmission delay, the CSM has not received the response from this CSP when the *cd* expires. In this situation, the CSM faces two challenges: (1) Since the CSM has only received *s*-1 responses, the aggregation of audit proofs cannot not complete. (2) The response delay is not intentionally caused by this positive CSP, so it is unfair to directly conclude that it is malicious. Thus, to ensure the feasibility in actual deployment, our proposal incorporates a fault-tolerant mechanism, that is, enabling probabilistic auditing. We denote the fault tolerance value (FTV) by *ξ*(1 ≤ *ξ* ≤ *n*), which also represents the number of audit proofs returned each time during the public auditing. But note that we will not focus on how to determine the FVT, which should be selected according to the actual deployment environment.

**Table 2:** The structure of SDT. The data structure of storage distribution table (SDT).

*RN*	*ID*	*SL*
1	*Fid*_1_	CSP_2_
2	*Fid*_2_	CSP_*l*_
…	…	…
*n*	*Fid*_*n*_	CSP_s_

### Result record table

Since CSPs are untrustworthy, if the results of each public auditing are gathered through probabilistic audit, the security of the proposal will be weakened. Therefore, the TPA should record the contents of each check in the result record table (RRT) stored locally, and its structure is shown in Table 3. *Chal* is an integrity challenge set generated by the TPA. Check result (CR) shows the audit result, and probability checking (PC) indicates whether it is a probabilistic verification; if so, the TPA needs write the FVT to the RRT.

**Table 3:** The structure of RRT. The data structure of result record table (RRT).

*chal*	CR	PC	FTV
*chal*_1_	1	0	None
*chal*_2_	1	1	FVT_2_
*chal*_3_	0	1	FVT_3_
*chal*_4_	0	0	None
…	…	…	

To be exact, there are four situations in RRT: {{*chal*, CR = 1, PC = 0, FVT = None}, {*chal*, CR = 1, PC = 1, FVT = *ξ*}, {*chal*, CR = 0, PC = 1, FVT = *ξ*}, {*chal*, CR = 0, PC = 0, FVT = None }}, and we give a detailed discussion.

(1) {*chal*, CR = 1, PC = 0, FVT = None}:means that the aggregated audit proof returned by the CSM has passed the TPA’s correctness verification, and this checking is not a probabilistic verification.
(2) {*chal*, CR = 1, PC = 1, FVT = *ξ*}: means that the aggregated audit proof returned by the CSM has passed the TPA’s correctness verification, but this checking is a probabilistic verification. The FVT indicates the number of CSPs participating in this public auditing.
(3) {*chal*, CR = 0, PC = 1, FVT = *ξ*} or {*chal*, CR = 0, PC = 0, FVT = None}: Since CR = 0, it means that the aggregated audit proof returned by the CSM has not passed the TPA’ correctness verification. The TPA terminates the check and immediately informs the Patient, and then the Patient runs the *Compensation* algorithm to claim compensation from the cloud service operator. And the highlighted part in Table 3 shows illegal situations.

**Remark 3:** Emphasize that for audit security, the number of consecutive CSM return probabilistic audit needs to be limited, for example: only 3 consecutive returns are allowed. That is, when the situation {*chal*, CR = 1, PC = 1, FVT = *ξ*} occurs in the RRT for the fourth time, the TPA will no longer proceed with the follow-up process and immediately inform the Patient.

**Remark 4:** The TPA will record the relevant information from each check in to the RRT in earnest, and the RRT can be reset at intervals during the actual deployment to save storage space.

In summary, in the public auditing phase, the TPA initiates integrity verification and dispatches the challenge message to the CSM. Then, the CSM transmits the challenge set *chal* to multiple CSPs according to the SDT and starts a countdown *cd*. Upon completion of the *cd*, the CSM calculates the aggregation audit proof *P*_*agg*_ based on the audit proofs and FVT replied by the CSPs. Following this, the TPA assesses whether to stop the correctness verification according to the FVT returned by the CSM. If affirmative, the TPA informs the Patient; if not, the TPA proceeds to verify the *P*_*agg*_’s correctness and updates the RRT. Regardless of the verification outcome, the TPA records all information in the RRT.

### The proposed scheme

A multi-replicas integrity checking scheme with supporting probability audit for cloud-based IoT are detailed introduced in this section.

### Setup

The PKG chooses two multiplicative cyclic groups *G*_1_ and *G*_2_ with prime order *p*, and *g* is a generator of *G*_1_. The Patient selects cryptographic hash functions: *H*, *H*_1_:0, 1^∗^ → *G*_1_ and the bilinear map *e*:*G*_1_ × *G*_1_ → *G*_2_. The PKG selects elements $x\in {Z}_{P}^{\ast }$, and computes the master secret key $msk={g}_{1}^{x}$ and master public key *mpk* = *g*^*x*^. The PKG randomly picks values *u*, *μ* ∈ *G*_1_, publishes the system public parameter *pp* = (*G*_1_, *G*_2_, *p*, *e*, *g*, *g*_1_, *H*, *H*_1_, *u*, *μ*, *mpk*) and holds the master secret key $msk={g}_{1}^{x}$ private.

### KeyGen

Upon receiving the key generation request from the Patient, the PKG performs the following operations: The PKG picks ${r}_{1}\in {Z}_{P}^{\ast }$ and computes *R*_1_ = *g*^*r*_1_^. The PKG calculates ${R}_{2}={g}_{1}^{x}\cdot (u\cdot H(ID))^{{r}_{1}}$ according to Patient’s identifier $ID\in {Z}_{P}^{\ast }$. And then the PKG returns *SK* = (*R*_1_, *R*_2_) to the Patient. After receiving the private key *SK*, the Patient verifies the correctness according to formula (1):

(1) \begin{eqnarray*}e({R}_{2},g)=^{{?}}e({g}_{1},mpk)\cdot e(u\cdot H(ID),{R}_{1}).\end{eqnarray*}

If (1) equation holds, accept; otherwise, reject it and inform to retransmit.

### ReplicaGen

To generate duplicate files, we utilize the symmetric encryption algorithm to obtain the encrypted file and then use PRP to obtain blinding factors corresponding to replica data blocks. That is, the Patient secretly chooses the encryption key *K*_1_ and the PRP key *K*_2_. Given that the outsourced file is *F*′, the Patient encrypts it using a symmetric encryption algorithm (AES, DES, *etc*.) denoted as *F* = *E*_*k*1_(*F*′). The Patient divides the encrypted file *F* into *m* blocks as $F={{b}_{j}^{{^{\prime}}}}_{1\leq j\leq m}$, where *m* represents the number of data blocks. For each data block ${b}_{j}^{{^{\prime}}}(1\leq j\leq m)$, the Patient calculates the blinding factor as *ϖ*_*ij*_ = *ψ*_*k*2_(*i*||*j*), where *i* represents the number of the duplicates and *ψ*_*k*2_ is the PRP with key *k*_2_, and computes the replica block as ${b}_{ij}={b}_{j}^{{^{\prime}}}+{\varpi }_{ij}$. Then, the Patient obtains the replica files as 𝔽 = *F*_*i*__1≤*i*≤*n*_ = *b*_*ij*__1≤*i*≤*n*,1≤*j*≤*m*_.

### TagGen

Since multiple copies are stored on different CSPs in our proposal, the tags set should be generated for each one. The Patient sets the replica identifier $Fi{d}_{i}\in {Z}_{P}^{\ast }$, computes *H*_1_(*Fid*_*i*_||*i*) and selects a random value ${r}_{2}\in {Z}_{P}^{\ast }$. For each block *b*_*ij*_(1 ≤ *i* ≤ *n*, 1 ≤ *j* ≤ *m*), the Patient computes tag as ${\sigma }_{ij}={R}_{2}\cdot ({H}_{1}(Fi{d}_{i}{|}{|}i)\cdot {\mu }^{{b}_{ij}})^{{r}_{2}}={g}_{1}^{x}\cdot (u\cdot H(ID))^{{r}_{1}}\cdot ({H}_{1}(Fi{d}_{i}{|}{|}i)\cdot {\mu }^{{b}_{ij}})^{{r}_{2}}$. Here, the tags set of each replica is denoted as *T*_*i*_ = *σ*_*ij*__1≤*i*≤*n*,1≤*j*≤*m*_ and all tags sets are 𝕋 = *T*_*i*__1≤*i*≤*n*_. The Patient sends duplicate file and tags sets 𝔽, 𝕋 to the CSM and deletes the local storage. Subsequently, the CSM check all *T*’s correctness, allocates CSP, records the allocation result in SDT, and uploads files.

### Challenge

The TPA periodically performs remote data integrity checking, records audit results in the RRT, and informs the Patient when necessary. The TPA picks a set *Q* with *c* elements, where *Q*⊆[1, *m*], and generates a set of random value ${v}_{j}\in {Z}_{P}^{\ast }$ for each *j* ∈ *Q*. Then the TPA sends *chal* = (*j*, *v*_*j*_)_*j*∈*Q*_ to the CSM. After receiving the message, the CSM searches the SDT, sends the integrity challenge *chal* = (*j*, *v*_*j*_)_*j*∈*Q*_ to the CSPs, and sets a countdown *cd*.

### ProofGen

Upon receiving the integrity challenge, the CSPs compute the block proof *λ*_*i*_ = ∑_(*j*,*v*_*j*_)∈*Q*_*v*_*j*_*b*_*ij*_ andthe tag proof ${\sigma }_{i}={\prod }_{(j,{v}_{j})\in Q}{\sigma }_{ij}^{{v}_{j}}$, and then reply to the CSM with the audit proofs *P*_*i*_ = *λ*_*i*_, *σ*_*i*__1≤*i*≤*n*_.

### ProofAgg

When the response from the CSPs is obtained, the CSM counts the number of audit proofs, updates FTV *ξ*, and aggregates the block proofs ${\lambda }_{agg}={\mathop{\sum }\nolimits }_{i=1}^{\xi }{\lambda }_{i}(1\leq i\leq \xi )$ andthe tag proofs ${\sigma }_{agg}={\mathop{\prod }\nolimits }_{i=1}^{\xi }{\sigma }_{i}(1\leq i\leq \xi )$. Then the CSM returns the aggregation audit proof *P*_*agg*_ = *λ*_*agg*_, *σ*_*agg*_ andFVT *ξ* to the TPA.

### ProofVerify

After receiving the response, the TPA looks for the FVT to assess if the FVT complies with the requirements. If the aggregation audit proof returned by the CSM is already the fourth probability verification, the TPA immediately informs the Patient and terminates the verification. Otherwise, the TPA continues and checks the correctness of the aggregation audit proof through Eq. (2):

(2) \begin{eqnarray*}e({\sigma }_{agg},g)& =& e({g}_{1},mpk)^{\sum _{(j,{v}_{j})\in Q}{v}_{j}}\cdot e(u\cdot H(ID),{g}^{{r}_{1}})^{\sum _{(j,{v}_{j})\in Q}{v}_{j}}\nonumber\\\displaystyle & & \cdot e \left( \prod _{i=1}^{\xi }({H}_{1}(Fi{d}_{i}{|}{|}i))^{\sum _{(j,{v}_{j})\in Q}{v}_{j}}\cdot {\mu }^{{\lambda }_{agg}},{g}^{{r}_{2}} \right) .\end{eqnarray*}

If (2) holds, returns ‘1’, which means that the integrity verification of duplicate files is successful and then the TPA records the *AR* and *PC* into the RRT. Otherwise, returns ‘0’, the TPA informs the Patient that the duplicate file was damaged, and the Patient runs the *Compensation* algorithm to claim compensation from the cloud service operator.

### Compensation

As mentioned above, the Patient runs this algorithm to claim against the cloud service operator when integrity checking fails.

**Remark 5:** Actually, when replica damage is detected, due to the blinding factor added to the *ReplicaGen* algorithm, a divide-and-conquer method can be used to recover bad blocks. The detailed process is no longer given here.

## Security analysis

**Theorem 1** **(Private key correctness):** The private key generated by the PKG will only be accepted after successfully passing the Patient’s correctness verification.

***Proof***: In *KeyGen* algorithm, after receiving the private key *SK*, the Patient verifies the correctness by checking the validity of formula (1):

\begin{eqnarray*}e({R}_{2},g)& =& e({g}_{1}^{x}\cdot (u\cdot H(ID))^{{r}_{1}},g) \nonumber\\\displaystyle & =& e({g}_{1}^{x},g)\cdot e((u\cdot H(ID))^{{r}_{1}},g) \nonumber\\\displaystyle & =& e({g}_{1},{g}^{x})\cdot e(u\cdot H(ID),{g}^{{r}_{1}}) \nonumber\\\displaystyle & =& e({g}_{1},mpk)\cdot e(u\cdot H(ID),{R}_{1}). \end{eqnarray*}

If Eq. (1) holds, the Patient accepts and uses it as the private key. Otherwise, reject it and inform to retransmit.

**Theorem 2 (Audit correctness):** Only when the CSPs correctly store the Patient’s replicas file, during the public auditing phase, the aggregation audit proof *P*_*agg*_ generated by the CSM can pass the TPA’s correctness verification.

***Proof***: In the *ProofVerify* algorithm, the TPA validates the correctness of the aggregation audit proof by checking the formula (2):

\begin{eqnarray*}e({\sigma }_{agg},g)& =& e \left( \prod _{i=1}^{\xi }{\sigma }_{i},g \right)  =e \left( \prod _{i=1}^{\xi } \left( \prod _{(j,{v}_{j})\in Q}{\sigma }_{ij}^{{v}_{j}} \right) ,g \right)  \nonumber\\\displaystyle & =& e \left( \prod _{i=1}^{\xi } \left( \prod _{(j,{v}_{j})\in Q}({R}_{2}\cdot ({H}_{1}(Fi{d}_{i}{|}{|}i)\cdot {\mu }^{{b}_{ij}})^{{r}_{2}})^{{v}_{j}} \right) ,g \right)  \nonumber\\\displaystyle & =& e \left( \prod _{i=1}^{\xi } \left( \prod _{(j,{v}_{j})\in Q}{R}_{2}^{{v}_{j}}\cdot (({H}_{1}(Fi{d}_{i}{|}{|}i)\cdot {\mu }^{{b}_{ij}})^{{r}_{2}})^{{v}_{j}} \right) ,g \right)  \nonumber\\\displaystyle & =& e \left( \prod _{i=1}^{\xi }\prod _{(j,{v}_{j})\in Q}{R}_{2}^{{v}_{j}}\cdot \prod _{i=1}^{\xi }\prod _{(j,{v}_{j})\in Q}({H}_{1}(Fi{d}_{i}{|}{|}i)\cdot {\mu }^{{b}_{ij}})^{{r}_{2}{v}_{j}},g \right)  \nonumber\\\displaystyle & =& e \left( \prod _{i=1}^{\xi }\prod _{(j,{v}_{j})\in Q}{R}_{2}^{{v}_{j}},g \right) \cdot e \left( \prod _{i=1}^{\xi }\prod _{(j,{v}_{j})\in Q}({H}_{1}(Fi{d}_{i}{|}{|}i)\cdot {\mu }^{{b}_{ij}})^{{r}_{2}{v}_{j}},g \right)  \nonumber\\\displaystyle & =& e \left( \prod _{(j,{v}_{j})\in Q}({g}_{1}^{x}\cdot (u\cdot H(ID))^{{r}_{1}})^{{v}_{j}},g \right) \cdot e \left( \prod _{i=1}^{\xi }\prod _{(j,{v}_{j})\in Q}({H}_{1}(Fi{d}_{i}{|}{|}i)\cdot {\mu }^{{b}_{ij}})^{{v}_{j}},{g}^{{r}_{2}} \right)  \nonumber\\\displaystyle & =& e \left( \prod _{(j,{v}_{j})\in Q}({g}_{1}^{x})^{{v}_{j}},g \right) \cdot e \left( \prod _{(j,{v}_{j})\in Q}(u\cdot H(ID))^{{v}_{j}},{g}^{{r}_{1}} \right) \nonumber\\\displaystyle & & \cdot e \left( \prod _{i=1}^{\xi } \left( \prod _{(j,{v}_{j})\in Q}({H}_{1}(Fi{d}_{i}{|}{|}i))^{{v}_{j}}\cdot \prod _{(j,{v}_{j})\in Q}{\mu }^{{b}_{ij}{v}_{j}} \right) ,{g}^{{r}_{2}} \right)  \nonumber\\\displaystyle & =& e \left( {g}_{1}^{\sum _{(j,{v}_{j})\in Q}{v}_{j}},{g}^{x} \right) \cdot e \left( (u\cdot H(ID))^{\sum _{(j,{v}_{j})\in Q}{v}_{j}},{g}^{{r}_{1}} \right) \nonumber\\\displaystyle & & \cdot e \left( \prod _{i=1}^{\xi }({H}_{1}(Fi{d}_{i}{|}{|}i))^{\sum _{(j,{v}_{j})\in Q}{v}_{j}}\cdot {\mu }^{\sum _{(j,{v}_{j})\in Q}{b}_{ij}{v}_{j}},{g}^{{r}_{2}} \right)  \nonumber\\\displaystyle & =& e({g}_{1},mpk)^{\sum _{(j,{v}_{j})\in Q}{v}_{j}}\cdot e(u\cdot H(ID),{g}^{{r}_{1}})^{\sum _{(j,{v}_{j})\in Q}{v}_{j}}\nonumber\\\displaystyle & & \cdot e \left( \prod _{i=1}^{\xi }({H}_{1}(Fi{d}_{i}{|}{|}i))^{\sum _{(j,{v}_{j})\in Q}{v}_{j}}\cdot \prod _{i=1}^{\xi }{\mu }^{{\lambda }_{i}},{g}^{{r}_{2}} \right)  \nonumber\\\displaystyle & =& e({g}_{1},mpk)^{\sum _{(j,{v}_{j})\in Q}{v}_{j}}\cdot e(u\cdot H(ID),{g}^{{r}_{1}})^{\sum _{(j,{v}_{j})\in Q}{v}_{j}}\nonumber\\\displaystyle & & \cdot e \left( \prod _{i=1}^{\xi }({H}_{1}(Fi{d}_{i}{|}{|}i))^{\sum _{(j,{v}_{j})\in Q}{v}_{j}}\cdot {\mu }^{\sum _{i=1}^{\xi }{\lambda }_{i}},{g}^{{r}_{2}} \right)  \nonumber\\\displaystyle & =& e({g}_{1},mpk)^{\sum _{(j,{v}_{j})\in Q}{v}_{j}}\cdot e(u\cdot H(ID),{g}^{{r}_{1}})^{\sum _{(j,{v}_{j})\in Q}{v}_{j}}\nonumber\\\displaystyle & & \cdot e \left( \prod _{i=1}^{\xi }({H}_{1}(Fi{d}_{i}{|}{|}i))^{\sum _{(j,{v}_{j})\in Q}{v}_{j}}\cdot {\mu }^{{\lambda }_{agg}},{g}^{{r}_{2}} \right) . \end{eqnarray*}

If (2) holds, it indicates that the integrity verification of duplicate files is successful; otherwise, the TPA informs the Patient.

**Theorem 3 (Resist forgery attack):** Our proposed scheme can effectively resist forgery attack.

### Proof

Suppose that the *l*th data block of the *κ*th replica has been corrupted, and this data block is just challenged by the TPA during the public auditing phase. As a result, the CSP is compelled to fabricate both the data block and its associated tag in an attempt to deceive the TPA’s verification. Denote the intact block and tag as (*b*_*κl*_, *σ*_*κl*_)_1≤*κ*≤*n*,1≤*l*≤*m*_, and the forged block and tag as $({\delta }_{\kappa l},{\sigma }_{\kappa l}^{{^{\prime}}})$. Note that, in accordance with the mathematical structure of the tag in our proposed scheme, the CSP can only fabricate the corresponding tag after successfully forging the data block. Then we proceed to analyze the probability that the CSP successfully forges both the data block and its corresponding tag.

### Analysis

(1) CSP forges data block

In the *ReplicaGen* phase, to obtain the encrypted file, our proposed scheme utilizes the symmetric encryption algorithm with the key ${K}_{1}\in {Z}_{P}^{\ast }$. And to obtain blinding factors *ϖ*_*κl*_ corresponding to replica data blocks, our proposed scheme uses PRP with the key ${K}_{2}\in {Z}_{P}^{\ast }$. So, it means that if the CSP can forge a valid data block *b*_*κl*_, it must be able to successfully guess *K*_1_ and *K*_2_ with non-negligible probability. But the probability of guessing that *K*_1_ and *K*_2_ are both 1/*p*. Furthermore, it implies that the probability of guessing *K*_1_ and *K*_2_ at the same time is 1/*p* × 1/*p* = 1/*p*^2^, which can be ignored since *p* is a large prime. Therefore, the probability that the CSP successfully forges a valid data block is 1/*p*^2^, which is negligible.

(2) CSP forges tag

After the CSP successfully guesses the block with the probability of 1/*p*^2^, it further attempts to forge the corresponding tag. The tag of valid data block *b*_*κl*_ is denoted as

(3) \begin{eqnarray*}{\sigma }_{\kappa l}={R}_{2}\cdot ({H}_{1}(Fi{d}_{i}{|}{|}i)\cdot {\mu }^{{b}_{\kappa l}})^{{r}_{2}}={g}_{1}^{x}\cdot (u\cdot H(ID))^{{r}_{1}}\cdot ({H}_{1}(Fi{d}_{i}{|}{|}i)\cdot {\mu }^{{b}_{\kappa l}})^{{r}_{2}}.\end{eqnarray*}

The tag of forged block *δ*_*κl*_ denoted as

(4) \begin{eqnarray*}{\sigma }_{\kappa l}^{{^{\prime}}}={R}_{2}^{{^{\prime}}}\cdot ({H}_{2}(Fi{d}_{i}{|}{|}i)\cdot {\mu }^{{\delta }_{\kappa l}})^{{r}_{2}}={g}_{1}^{{x}^{{^{\prime}}}}\cdot (u\cdot H(ID))^{{r}_{1}}\cdot ({H}_{2}(Fi{d}_{i}{|}{|}i)\cdot {\mu }^{{\delta }_{\kappa l}})^{{r}_{2}}.\end{eqnarray*}

If the CSP can successfully forge the tag, then formula (3) is equal to formula (4), and we have the following:

(5) \begin{eqnarray*}1= \frac{{\sigma }_{ij}}{{\sigma }_{ij}^{{^{\prime}}}} = \frac{{g}_{1}^{x}\cdot (u\cdot H(ID))^{{r}_{1}}\cdot ({H}_{1}(Fi{d}_{i}{|}{|}i)\cdot {\mu }^{{b}_{ij}})^{{r}_{2}}}{{g}_{1}^{{x}^{{^{\prime}}}}\cdot (u\cdot H(ID))^{{r}_{1}}\cdot ({H}_{1}(Fi{d}_{i}{|}{|}i)\cdot {\mu }^{{\delta }_{ij}})^{{r}_{2}}} = \frac{{g}_{1}^{x}\cdot {{\mu }^{{b}_{ij}}}^{{r}_{2}}}{{g}_{1}^{{x}^{{^{\prime}}}}\cdot {{\mu }^{{\delta }_{ij}}}^{{r}_{2}}} .\end{eqnarray*}

If Eq. (5) holds, it can infer that ${g}_{1}^{x}\cdot {{\mu }^{{b}_{ij}}}^{{r}_{2}}={g}_{1}^{{x}^{{^{\prime}}}}\cdot {{\mu }^{{\delta }_{ij}}}^{{r}_{2}}$ holds and further, it means that ${g}_{1}^{x}={g}_{1}^{{x}^{{^{\prime}}}}$ holds. Actually, in the *Setup* phase, we know that $x\in {Z}_{P}^{\ast }$ is randomly picked by the PKG, and ${g}_{1}^{x}$ is the master secret key that its mathematical structure and content are both kept secret and known only to the PKG. Since the PKG is the trusted entity, it means that ${g}_{1}^{x}$ is not forgeable. That is, even if the CSP can successfully forge a valid data block with the probability of 1/*p*^2^, it cannot forge the corresponding tag.

Thus, from the above series of analysis, it can be seen that our proposed scheme can effectively resist the forgery attack.

**Theorem 4 (Resist replace attack):** Our proposed scheme can effectively resist the forgery attack.

### Proof

Suppose that the *l*th data block of the *κ*th replica has been corrupted, and this data block is just challenged by the TPA during the public auditing phase. Therefore, the CSP replaces the challenged data block and tag with another in an attempt to pass the TPA’s verification. The analysis is similar to Theorem 3, so is omitted here.

**Theorem 5 (Resist replay attack):** Our proposed scheme can effectively resist forgery attack.

### Proof

Suppose that the *l*th data block of the *κ*th replica has been corrupted, and this data block is just challenged by the TPA during the public auditing phase. Therefore, the CSP returns the audit proof that has been previously checked in an attempt to pass the TPA’s verification. The analysis is similar to Theorem 3, so is omitted here.

## Results

### Quantitative analysis and comparison

We first define the symbols used and their meanings as shown in Table 4. To be fair, we set the number of data sectors to 1. Here, we no longer take the addition and PRP operation into account, because they are time-saving in actual deployment. Note that *δ* represents the fault tolerance value and index the number of audit proofs collected during the audit process.

**Table 4:** Notations and meanings. Notations and meanings used in quantitative analysis and comparison.

**Notation**	**Meaning**	**Notation**	**Meaning**
*H*	One hash operation	*n*	The number of replica file
*Mul*	One multiplication operation	*m*	The number of data block
*P*	One pair operation	*δ*	*FVT*
*Exp*	One exponentiation operation	*c*	The number of the challenged block
*E*	One encryption operation	|*p*|	The size of an element in ${Z}_{P}^{\ast }$ or *G*_1_
|*q*|	The size of an element in *Z*_*q*_	–	–

### Computation overhead

The computational overhead comparison with the scheme (Li, Yan & Zhang, 2021) is shown in Table 5. The process begins with the Patient encrypting the outsourced file, dividing the data blocks, and applying a random mask for blind operation. This entails time-saving PRP and addition operations, which can be ignored. That is, the overhead of the *ReplicaGen* stage amounts to just one encryption operation. To generate the tag set, the Patient sets the replica identifier *F*_*id*_ and executes the hash operation, and then calculates the tag for each block. As a result, the total computational overhead of the *TagGen* stage is denoted by *mnH* + 2*mnMul* + 2*mnExp*. In the *ProofGen* phase, the CSPs compute and return the block proof *λ*_*i*_ and tag proof *σ*_*i*_ to CSM. Among them, the calculation of *λ*_*i*_ requires *c* multiplication operations, and the calculation of *σ*_*i*_ requires *nc* exponentiation operation and *n* (2*c*-1) multiplication operations, so the total overhead is *ncExp* + *n*(2*c* − 1)*Mul*. During the *ProofAgg* phase, the CSM aggregated audit proof according to the number of block proofs and tag proofs returned by the CSPs. Since the time-consuming addition operation is not considered, the cost is (*ξ* − 1)*Mul*. To verify the correctness of the aggregation audit proof, in the *ProofVerify* phase, the TPA leverages FVT to check whether formula (2) holds and the overall calculation cost is 5*P* + (*ξ* + 1)*H* + (*ξ* + 3)*Exp* + (*ξ* + 1)*Mul*.

**Table 5:** Computational overhead comparison.

Schemes	Phase				
	ReplicaGen	TagGen	ProofGen	ProofAgg	ProofVerify
Scheme (Li, Yan & Zhang, 2021)	*nE*	*mnH* + 3*mnMul* + 2*mnExp*	*cExp* + (*cn* + *c* − 1)*Mul*	*nMul*	3*P* + (*nc* + 1)*H* + 2(*nc* + 1)*Mul* + *ncExp*
Our scheme	*E*	*mnH* + 2*mnMul* + 2*mnExp*	*ncExp* + *n*(2*c* − 1)*Mul*	(*ξ* − 1)*Mul*	5*P* + (*ξ* + 1)*H* + (*ξ* + 3)*Exp* + (*ξ* + 1)*Mul*

### Communication overhead

Table 6 presents a comparison of the communication costs incurred in three stages between our proposal and the scheme (Li, Yan & Zhang, 2021). Note that the data fragmentation technique is employed in Scheme (Li, Yan & Zhang, 2021), so *s* represents the number of the data sector. During the integrity challenge phase, the CSM sends *chal* to *n* CSPs. Since each challenge occupies *c*(|*p*| + |*q*|), the communication cost for this phase is *cn*(|*p*| + |*q*|). After receiving the verification tasks, the CSPs compute and return the aduit proof. Due to the adoption of probabilistic audting, the number of aduit proofs returned is denoted as *ξ*, and this phase incurs the communication cost of 2*ξ*|*p*| bits. The CSM sends the aggregation audit proof along with FVT to the TPA, resulting in a total communication cost of 3|*p*| bits. From Table 6, it can be observed that the communication costs incurred in all three stages of our scheme are lower than those of the scheme (Li, Yan & Zhang, 2021).

**Table 6:** Communication overhead comparison.

Schemes	Challenge	Response from CSPs	Response to TPA
Scheme (Li, Yan & Zhang, 2021)	*n*(3|*p*| + log2*c*)	*ξ*(|*p*| + *s*|*p*|)	(3 + 2*s*)|*p*|
Our scheme	*cn*(|*p*| + |*q*|)	2*ξ*|*p*|	3|*p*|

### Experiments

We run a series of experiments on the 2.80 GHz Intel Core i7 processor and 16.0GB RAM. All the experiments use the Type A with the free Pairing-Based Cryptography (PBC) Library. In the implementation, we selected the file “a.param” as the parameter for the free Pairing- Based Cryptography (PBC) Library. In the experiment, we created a 4M sized data file, with each data block set to a size of 4KB.

**Figure 3:**
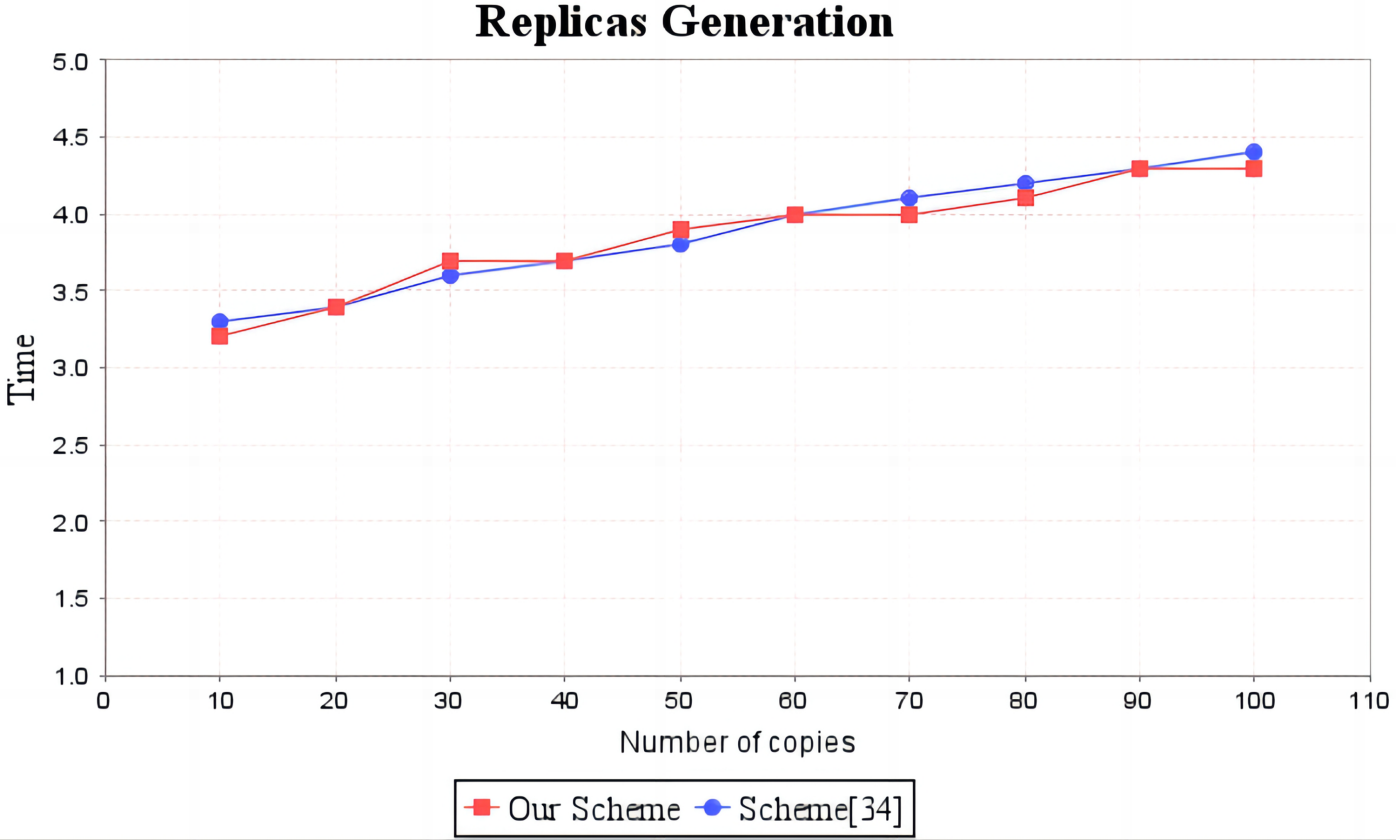
Computation cost of replicas generation. The red line shows the change trend of replica generation time of our scheme as the number of replicas increases. The blue line shows the change trend of the copy generation time of scheme (Li, Yan & Zhang, 2021) as the number of copies increases.

**Figure 4:**
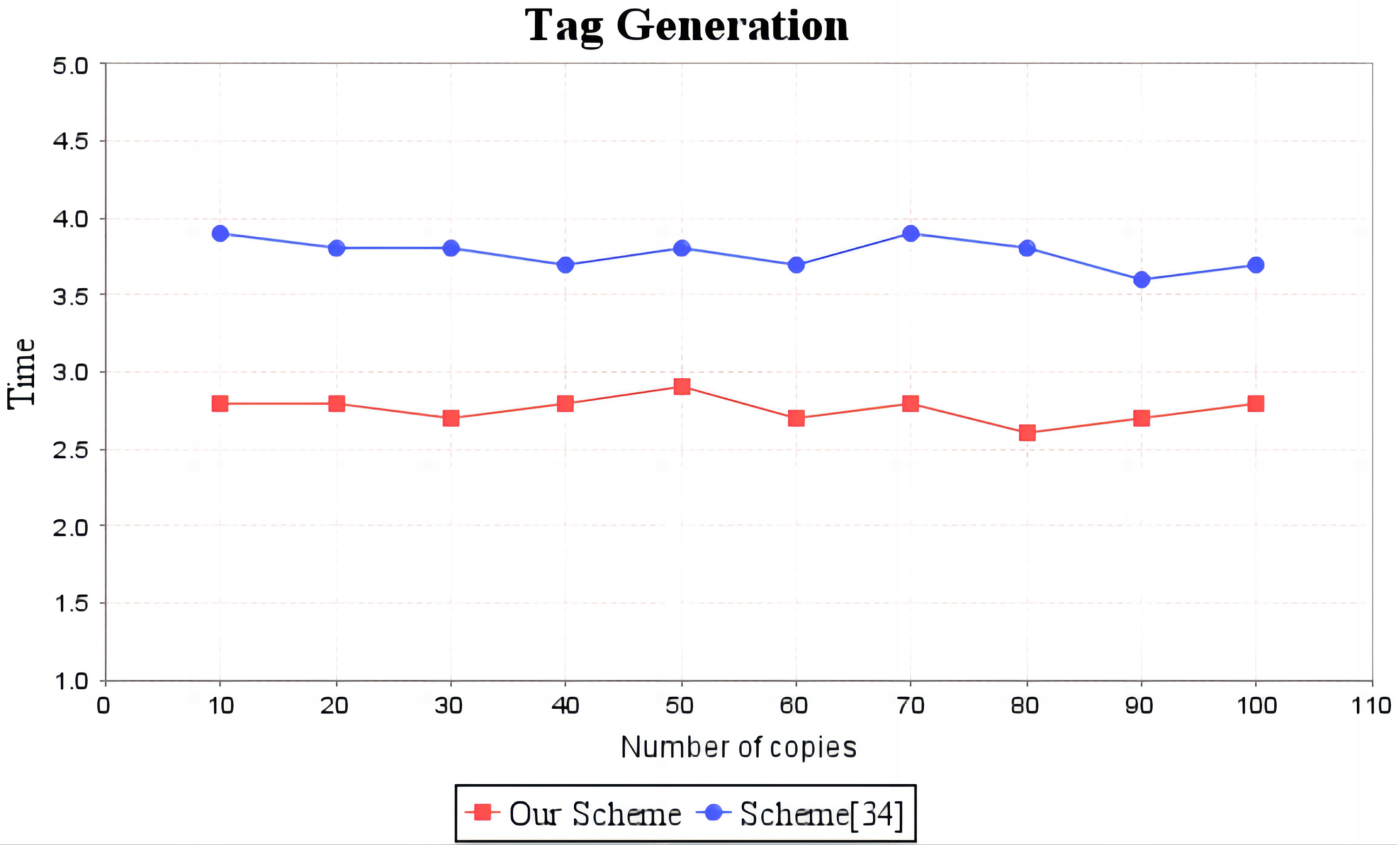
Computation cost of tag generation. The red line shows the trend of tag generation time of our scheme as the number of replicas increases. The blue line shows the change trend of the tag generation time of scheme (Li, Yan & Zhang, 2021) as the number of replicas increases.

**Figure 5:**
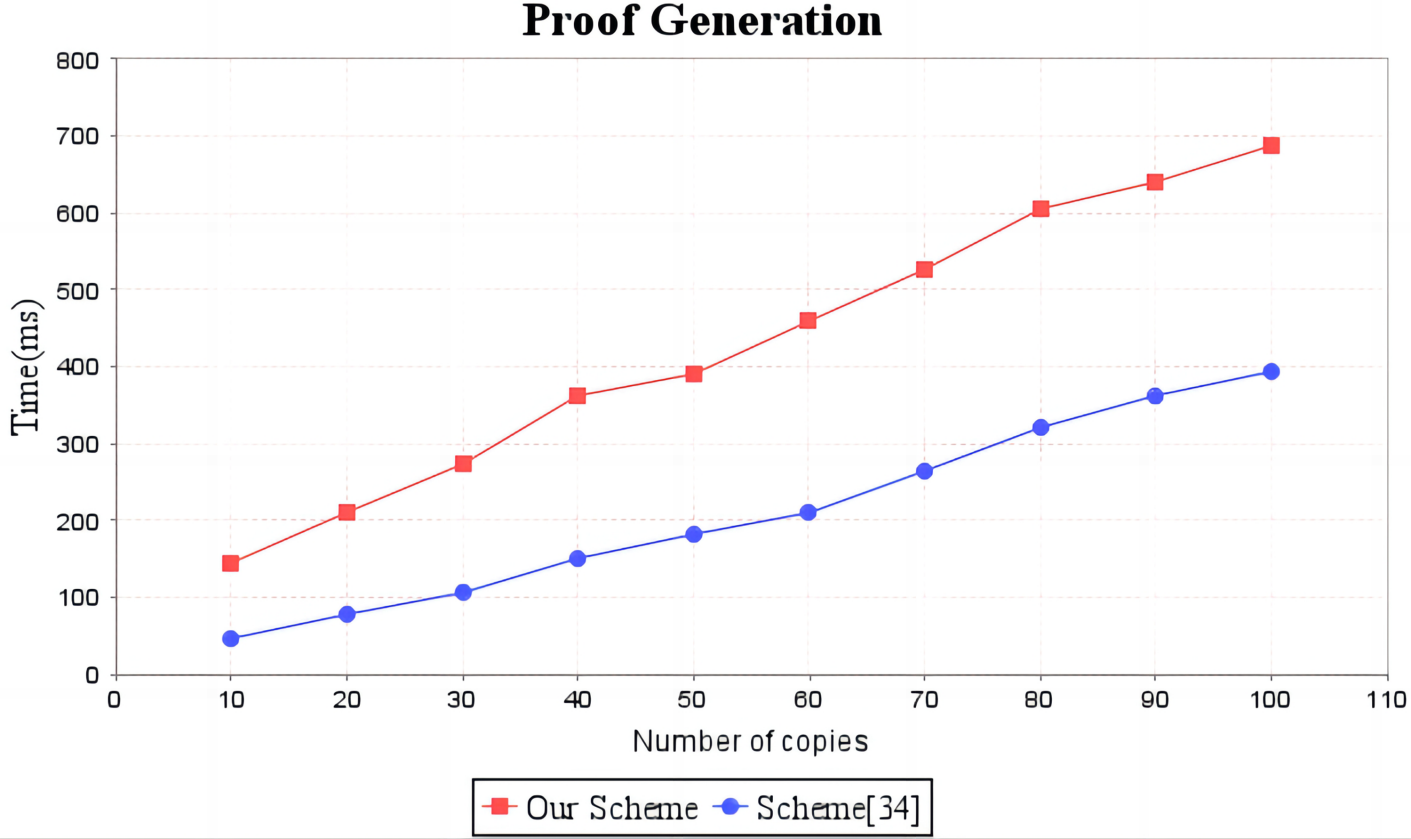
Computation cost of proof generation. The red line shows the trend of audit proof generation time in our scheme as the number of replicas increases. The blue line shows the change trend of the audit proof generation time of scheme (Li, Yan & Zhang, 2021) with the increase of the number of replicas.

**Figure 6:**
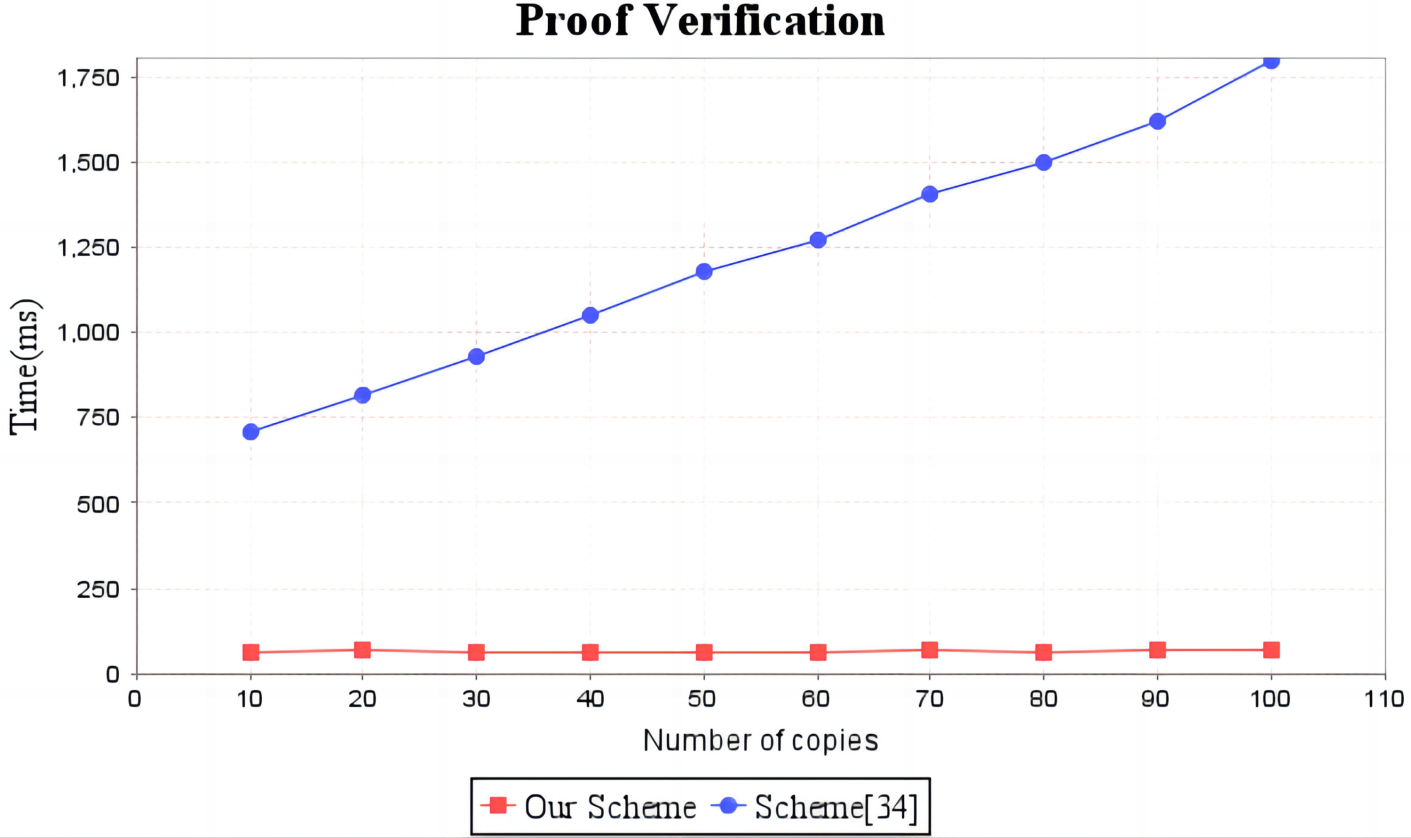
Computation cost of proof verification. The red line shows the trend of audit proof verification time of our scheme as the number of replicas increases. The blue line shows the change trend of the audit proof verification time of scheme (Li, Yan & Zhang, 2021) with the increase of the number of copies.

In the experimental section, a series of comparisons were conducted between the scheme (Li, Yan & Zhang, 2021) and our proposal. It can be seen from Fig. 3 that the cost incurred in the replica generation stage is similar for both schemes, increasing linearly with the number of replicas between 3s and 4.5s. Figure 4 illustrates that the time required to generate the tag set does not increase with the number of replicas for both schemes, but our proposal requires significantly less time than the scheme (Li, Yan & Zhang, 2021). In the audit proof generation phase, as shown in Fig. 5, our scheme takes more time due to the expensive modular exponentiation operations compared to the scheme (Li, Yan & Zhang, 2021). In the audit proof verification stage, as shown in Fig. 6, the scheme (Li, Yan & Zhang, 2021) exhibits significantly higher time consumption than our proposal, which is consistent with the analysis results in Table 5.

## Discussion

This article proposes a multi-replica integrity verification scheme that supports probabilistic auditing, taking into account the context of the Internet of Things (IoT) and shared healthcare. The article begins by analyzing critical issues in existing multi-replica integrity verification schemes. The proposed scheme aims to address the problem of synchronization verification of EHR replica files on CSPs located in different geographical locations. We introduce a novel approach called probabilistic auditing, and based on IBE, we generate private keys and construct an HVT, effectively avoiding the overhead of using public key certificates. Under the CDH assumption, the proposed scheme has been proven to be secure and can effectively withstand forgery, replace, and replay attacks. Theoretical analysis and experimental results demonstrate the efficiency and practicality of our scheme. However, when verifying the integrity of replicas on different CSPs, there will inevitably be a trade-off between accuracy and computational or communication costs. In future work, we will focus on addressing this issue and identify effective measures to strike a balance between cost and efficiency.

## Additional Information and Declarations

### Data Availability

The following information was supplied regarding data availability: The code is available at Zenodo: Yang. (2023). yf0219/Multi-replica-experiment-of-Cloud-storage: First release (code). Zenodo. https://doi.org/10.5281/zenodo.10117378.
